# The Positive Spiral Between Problem-Solving Management and Trust: A Study in Organizations for Individuals With Intellectual Disability

**DOI:** 10.3389/fpsyg.2020.617622

**Published:** 2021-02-04

**Authors:** Yolanda Estreder, Vicente Martínez-Tur, Inés Tomás, Alice Maniezki, José Ramos, Luminiţa Pătraş

**Affiliations:** ^1^IDOCAL-University of Valencia, Valencia, Spain; ^2^IDOCAL-University of Valencia and IVIE, Valencia, Spain

**Keywords:** problem-solving, conflict management, trust, organizations for individuals with intellectual disability, professionals, families, dynamic, reciprocal

## Abstract

To achieve their goals, organizations for individuals with intellectual disability have to stimulate high-quality relationships between professionals and family members. Therefore, achieving professionals’ trust in family members has become a challenge. One relevant factor in explaining professional’s trust in families is the degree to which family members use the “problem-solving” conflict management strategy (high concern for oneself but also for the other party) in their disputes–disagreements with professionals. It is reasonable to argue that when family members use problem-solving conflict management, professionals’ trust increases. Professionals’ trust, in turn, stimulates the use of problem-solving strategies by family members. However, it is also plausible that professionals are the initiators of this positive spiral (professionals’ trust–problem-solving conflict management by family members–professionals’ trust). To examine this relationship between problem solving and trust over time, we conducted a longitudinal survey study in which 329 professionals reported on these two constructs three times (with 4 weeks between the measurements). Using structural equation modeling, we compared four nested models: (a) stability, (b) causality (where the problem-solving strategy by familiar members is the initiator of the spiral), (c) reversed causation (where the professional’s trust is the initiator of the spiral), and (d) reciprocal (where problem-solving conflict management and trust reinforce each other). The results of the χ^2^ difference tests, regarding the comparison of the models, showed that the reciprocal model was significantly superior to the alternative proposals. Our findings supported a complex view of the relationships between problem-solving conflict management and trust, based on dynamic reciprocal relationships over time.

## Introduction

This study was carried out in organizations for individuals with intellectual disability (IID). The main goal in this context is to enhance quality of life of service users. To do so, fruitful partnerships between professionals and family members are crucial (Turnbull et al., 2006; Colarusso and O’Rourke, 2007). This is especially evident in this context because a long-term relationship between professionals and family members usually exists (Molina et al., 2015), and organizational actions are important for the lives of service users. Therefore, understanding and stimulating cooperation and trust is a relevant objective in this type of organization (Mereoiu et al., 2016). With this in mind, we examine the dynamic relationships over time between professionals’ perceptions of the degree to which family members use problem-solving conflict strategies, on the one hand, and professionals’ trust in family members, on the other.

Conflicts are pervasive because they are potentially present in all the different facets of organizational life (Caesens et al., 2019). According to research on effectiveness (Langfred, 2007; Curşeu and Schruijer, 2010), trust is able to reduce conflict and, therefore, positively influence effectiveness (Peterson and Behfar, 2003). Scholars have also argued that disputes can have positive effects if they are managed adequately (Rahim and Mager, 1995; Elsayed-Ekhouly and Buda, 1996; Hempel et al., 2009). Hence, we argue that research should not only focus on the undeniable existence of conflict within organizations but also on the possibility of managing these conflicts in a constructive way. With this in mind, conflict management and trust have become two crucial related constructs in organizations because they help to understand and build constructive relationships (see Elgoibar et al., 2016). Based on the Theory of Cooperation and Competition (Deutsch, 1973) and Dual Concern Theory (Pruitt and Rubin, 1986; De Dreu et al., 2001), different conflict management strategies have been proposed that combine concerns for the self and for others in different ways. Of these strategies, “problem-solving” is especially useful for building constructive relationships because it is oriented toward satisfying the aspirations of both parties (Williams, 2001). For this reason, we concentrate on problem-solving conflict management. The use of this type of strategy should enhance trust because it demonstrates a common sense of belonging (Hempel et al., 2009), communicating the intention to manage the conflict in a cooperative and mutually beneficial way (Wong et al., 2019). Accordingly, problem-solving conflict management and trust should be intimately associated because trust is an indicator of a constructive relationship between the parties, typically defined as the acceptance of vulnerability during social interactions, based on the expectation of others’ benevolent motives (Rousseau et al., 1998). Experimental studies have also investigated how trust and cooperative behaviors are connected by using social dilemmas (see Cook and Cooper, 2003; Ostrom and Walker, 2003). In this framework, the links from trust to cooperation are persistent, but some factors have a significant influence on this relationship (Balliet and Van Lange, 2013). In their meta-analysis, these authors found that the relationship was stronger for larger conflicts (compared to smaller), and individual interactions (compared to intergroup). Other studies have also observed that situational (e.g., time pressure) and individual differences (e.g., personality) contribute to the selection of competitive vs. cooperative strategies (Kuzmicheva, 2020).

Although research has generally supported the link from conflict management based on cooperative problem-solving to trust (see Elgoibar et al., 2016), the nature of their mutual relationships over time remains unexplored. As in other areas of organizational psychology (e.g., organizational climate), there is discussion about the role of the context and the individual (see Schneider, 1987). That is, there is a debate about who is the initiator of change processes. In the relationship between conflict management and trust, the idea that the context (e.g., how the other party manages conflicts) influences individual judgments (trust) predominates. Nevertheless, individuals are also able to impact the organizational context. In fact, although it is generally accepted that cooperative conflict management leads to trust (e.g., Hempel et al., 2009; Yang, 2012; Sahoo and Sahoo, 2019; Wong et al., 2019), some researchers have proposed the opposite direction, confirming that trust is a strong precursor of using cooperative conflict management (Holtgrave et al., 2020). With this ambiguity in mind, we contribute to the literature on conflict management and trust in three ways. First, we attempt to create a consensus by clarifying the direction of the relationship between the two constructs by testing four alternatives: stability, causality (where problem-solving conflict management is the starting point and leads to subsequent trust), reversed causation (where trust is the starting point and contributes to subsequent problem-solving conflict management), and reciprocal influence (where problem-solving conflict management and trust reinforce each other). Clarifying the direction of the relationship is relevant because it contributes knowledge about how conflict management and trust operate in organizations. Previous efforts considered the interplay between trust and the type of conflict (Curşeu and Schruijer, 2010), but not strategies to manage conflicts. Although factors such as the type and intensity of the conflict are relevant, conflict management more directly addresses the way conflicts are dealt with, which is fundamental for organizations (Tjosvold, 1998; De Dreu et al., 2001), and requires considerable effort from their members (Thomas, 1992). Through an in-depth study of the nature of the relationship, we hope to gain insight into the development of two constructs with strong potential as suitable factors for understanding organizational effectiveness. Second, we address calls for research on the dynamics of the relationship between conflict management and trust (García et al., 2016; Tjosvold et al., 2016). The consideration of temporal dynamics is critical for theory building in organizational psychology, providing a more accurate view of phenomena (George and Jones, 2000; Roe, 2008). Accordingly, we measure both problem-solving conflict management and trust at three time points, which makes it possible to test their interrelations over time. Finally, we answer calls for trust research that considers domains other than vertical relations in organizations (Ferres et al., 2004; Han and Harms, 2010). To do so, we focus on professionals working in organizations for IID. This is a unique organizational setting where professionals usually establish long-term relationships with family members as service users, establishing strong emotional bonds. In fact, previous evidence in organizations for IID confirmed that professionals and family members interact for many years. Employees work in the organization and family members use the services for at least 6 to 10 years, on average (Molina et al., 2015; Moliner et al., 2017). Although cooperation between professionals and parents is necessary to achieve organizational goals (i.e., improve quality of life of IID; Mereoiu et al., 2016), disagreements often arise (Deslandes et al., 1999; Zhang et al., 2002; Martínez-Tur et al., 2018), and enhancing professionals’ trust in families has become an important challenge (Turnbull and Turnbull, 2015). With this in mind, our research focuses on problem solving because this specific conflict management strategy is suitable for the unique research context addressed. When family members and professionals cooperate, managing a conflict in a way that is beneficial to both parties seems appropriate. Their cooperation is necessary, not only to reach specific task objectives in the service for the IID but also to establish and nourish their relationship (Molina et al., 2015), which then fosters the support system itself and guarantees the continuing functionality of the service.

We expect the use of problem-solving conflict management by family members of IID to be positively related to professionals’ trust in families. In turn, we also expect professionals’ trust to stimulate the use of problem-solving conflict management by family members. In the following paragraphs, we describe in greater detail the three aforementioned models (causality, reversed causation, and reciprocal) that connect problem-solving conflict management and trust over time.

In the present study, the *causality model* refers to the predominant view that problem-solving conflict management contributes to subsequent trust. As mentioned above, there is consistent empirical evidence supporting this link from problem-solving to trust. Based on the Social Identity Theory, Hempel et al. (2009) argued that when one of the parties in a relationship uses problem-solving strategies to manage conflicts, this party informs the other about the existence of a common sense of belonging and identity where both parties’ aspirations are respected. As a result of this shared identity, the other party perceives benevolence in the treatment and is willing to be vulnerable; in other words, trust emerges. We transfer this argument to the social interactions between professionals and family members in organizations for IID. When professionals perceive that family members use problem-solving strategies, professionals’ trust in family members increases.

In the *reversed causation model*, we refer to a proposal that changes the order in the sequence. According to this model, trust facilitates the use of problem-solving conflict management. Holtgrave et al. (2020) suggested that when people face a conflict, they always develop attributions about others’ trustworthiness, regardless of whether this expectation is accurate. This initial level of trust influences the use of conflict management strategies. The other party reciprocates by showing cooperative conflict management based on problem solving. By extending this rationale to our research context, it is reasonable to argue that professionals who trust in families will stimulate reciprocation, and therefore, family members will use problem-solving management strategies in their interactions with professionals.

The combination of the causality and reversed causation proposals makes it possible to formulate the *reciprocal model*, characterized as a cycle or spiral. In this model, temporal dynamics play a significant role. This proposal is not incompatible with the two aforementioned models. In fact, the reciprocal model combines both arguments by proposing that problem-solving conflict management and trust reinforce each other. Regardless of the starting construct, there is an interrelation between problem-solving conflict management and trust over time. The use of problem-solving conflict management (T1) stimulates subsequent trust (T2), and trust, in turn, enhances the use of problem-solving conflict management (T3). In the same way, initial trust (T1) leads to subsequent problem-solving conflict management (T2), and the use of this strategy, in turn, increases trust (T3). To capture this positive spiral, professionals working in organizations for IID reported their trust in families and the use of problem-solving conflict management by family members at three separate measurement times.

Based on these arguments, we empirically examine three competing hypotheses. Testing competing hypotheses helps to avoid a traditional myopia in science that focuses on finding evidence to support a certain hypothesis while disregarding evidence that supports alternative results (Nuzzo, 2015).

Hypothesis 1: Perceptions that family members use problem-solving conflict management will increase professionals’ subsequent trust in family members.
Hypothesis 2: Professionals’ trust in family members will increase family members’ subsequent use of problem-solving conflict management.
Hypothesis 3: The use of problem-solving conflict management by family members and professionals’ trust in families will have positive reciprocal relations over time.

## Materials and Methods

### Participants

Professionals participating in the current research study were working in 59 small centers dedicated to the attention of IID. All these centers were affiliated with “Plena inclusión,” a Spanish non-governmental organization dedicated to improving the quality of life of this vulnerable group. Each center agreed to recruit a small number of professionals, so that the longitudinal data collection did not interfere with the operation of the center in question. Professionals were randomly selected from those who had direct interaction with family members as part of their daily work. This sampling plan resulted in a response rate above 90%. A total of 406 professionals answered our questionnaire at T1, but 77 declined to participate in T2, or T3. Therefore, the final study sample was composed of 329 professionals (81%). About 76% of them were women, and they were 39.12 (SD = 0.92) years old on average, with a mean tenure of 11.24 (SD = 7.57) years working in the organization. We compared this sample with professionals who answered in T1 but did not continue in the two subsequent assessments (*N* = 77). Differences in the distribution of women vs. men [χ^2^_(_*_1_*_)_ = 2.20, *p* > 0.05] and age [*t*_(_*_392_*_)_ = −0.77, *p* > 0.05] were not statistically significant. Furthermore, differences in their tenure were not significant [*t*_(_*_358_*_)_ = −0.68, *p* > 0.05]. These results indicate that our final sample was not biased.

### Procedure

The Ethical Committee of Research in Humans of the university of the corresponding author approved the current research study. Researchers from this university contacted “Plena inclusión” to explain the objectives and procedure of the research study. Once this organization had agreed to participate in the project, we trained one employee per center. We explained how to select participants randomly and correctly carry out the data collection, respecting the temporal distance between measurement times. Main concepts were described, as well as the meaning of informed consent and how to establish fluid communication between the centers and the research team. The professionals who were instructed in the training sessions did not participate in the study. Participating professionals signed an informed consent where voluntariness and confidentiality were ensured. They were informed about the objectives and procedure of the project, and they were allowed to leave the study at any time. Participants selected were professionals who had daily contact with family members. They answered our questionnaire three times with a separation of 4 weeks.

### Measures

To measure *problem-solving conflict management*, we used the validated four-item scale by De Dreu et al. (2001), with Likert response options ranging from 1 (*never*) to 5 (*always*). The wording of the items was adapted to the context of professionals working in organizations for IID and interacting with family members (e.g., “Family members examine ideas from both sides to find a mutually optimal solution”). Regarding professionals’ *trust* in family members, we used the four-item general trust scale by Butler (1991; e.g., “I feel I can trust the family members of this center”) with Likert response options ranging from 1 (*strongly disagree*) to 5 (*strongly agree*). For these two measures, we compared scores of the final sample with scores of professionals who answered in T1 but did not continue in T2 or/and T3 (*N* = 77). There were no significant differences in problem-solving conflict management [*t*_(_*_392_*_)_ = −0.65, *p* > 0.05] or trust [*t*_(_*_392_*_)_ = −0.74, *p* > 0.05]. Again, this result indicates that our final sample was not biased.

### Statistical Analyses

To test our hypotheses, we conducted structural equation modeling (SEM) methods using MPlus (Muthén and Muthén, 1998-2010). Items on the corresponding scales were introduced as observed variables, and relationships between latent variables were modeled. Item distribution was tested. Considering that skewness and kurtosis coefficients were in the range between −1 and 1, approximation to normality was supported (Muthén and Kaplan, 1985, 1992). Because our items had sufficient response categories (five response categories) and reasonably met the normality assumption, maximum likelihood (ML) was used as the estimation method (Lloret-Segura et al., 2014, 2017). Four nested models that included six latent variables (problem solving and trust at T1, T2, and T3) were performed to compare a series of cross-lagged models using the χ^2^ difference test (Jöreskog and Sörbom, 1993). First, following the procedure by Demerouti et al. (2004), we tested a model (Model 1) that included the temporal stabilities (each variable receives the effect of that same variable in a previous time, e.g., T3 trust regressed on T2 and T1 trust, T2 trust regressed on T1 trust) and synchronous correlations (modeling correlations between the variables for each possible pair of measurement waves, e.g., T1 trust correlated with T1 problem solving, T2 trust correlated with T2 problem solving, and T3 trust correlated with T3 problem solving). Second, we tested the causality model (Model 2), which is identical to Model 1 but also includes cross-lagged pathways from problem solving at Time 1 (T1) to trust at T2 and T3, respectively, as well as from problem-solving conflict management at T2 to trust at T3 (Hypothesis 1). Third, we tested the reversed causation model (Model 3), which is identical to Model 1 but also includes cross-lagged pathways from trust at T1 to problem-solving conflict management at T2 and T3, respectively, as well as from trust at T2 to problem-solving conflict management at T3 (Hypothesis 2). Finally, we tested the reciprocal model (Model 4), which is identical to Model 1 but also includes reciprocal relationships between problem-solving conflict management and trust (all paths from Models 2 and 3; Hypothesis 3). Additionally, in all the aforementioned models, the covariance between measurement errors of the same item at different time points was specified. Model 1 was compared with three proposed models (Models 2, 3, and 4) that include our hypotheses. Additionally, Models 2 and 3 were compared with Model 4. The regression coefficients were assessed using one-tailed tests, which are appropriate for directional hypotheses (Erickson and Nosanchuk, 1977; Wonnacott and Wonnacott, 1984).

## Results

### Preliminary Results

Means, standard deviations, correlations, and reliability estimates among variables are presented in Table 1. As expected, the results revealed significant positive correlations between all the assessed variables. The scales had good reliability properties, ranging from 0.83 to 0.94.

**TABLE 1:** Descriptive statistics, reliability, and correlations among the study variables.

	Range	Mean	SD	1	2	3	4	5	6
1. Problem solving T1	1–5	3.37	0.76	(0.90)					
2. Trust T1	1–5	3.77	0.66	0.38**	(0.83)				
3. Problem solving T2	1–5	3.42	0.78	0.50**	0.33**	(0.93)			
4. Trust T2	1–5	3.80	0.69	0.39**	0.52**	0.50**	(0.86)		
5. Problem solving T3	1–5	3.43	0.78	0.53**	0.37**	0.67**	0.54**	(0.94)	
6. Trust T3	1–5	3.78	0.66	0.35**	0.56**	0.43**	0.67**	0.54**	(0.86)

T1, Time 1; T2, Time 2; and T3, Time 3. Pearson’s correlation coefficient was computed. Cronbach’s alpha coefficients appear on the diagonal in brackets; ** *p* < 0.01.

We conducted confirmatory factor analysis (CFA) to confirm the distinctiveness of the two study variables (problem solving and trust). ML was chosen as the estimation method because all the items followed a normal distribution. We tested two nested competing models: (1) a two-factor model identifying the items on the two separate scales and (2) a one-factor model where problem-solving and trust items were combined into a single factor. We examined these two competing models for the variables at the three time measurement points. The theorized two-factor model fit the data well at T1[χ^2^_(19)_ = 50.680, *p* < 0.01, CFI = 0.981, TLI = 0.972, and RMSEA = 0.071], T2 [χ^2^_(19)_ = 43.706, *p* < 0.01, CFI = 0.988, TLI = 0.982, and RMSEA = 0.063], and T3 [χ^2^_(19)_ = 38.346, *p* < 0.01, CFI = 0.991, TLI = 0.986, and RMSEA = 0.056]. By contrast, the one-factor model showed worse fit at T1 [χ^2^_(20)_ = 766.199, *p* < 0.01, CFI = 0.553, TLI = 0.374, and RMSEA = 0.337], T2 [χ^2^_(20)_ = 706.975, *p* < 0.01, CFI = 0.662, TLI = 0.526, and RMSEA = 0.324], and T3 [χ^2^_(20)_ = 566.429, *p* < 0.01, CFI = 0.733, TLI = 0.626, and RMSEA = 0.289]. The chi-square difference between the two-factor and one-factor models at T1 (χ^2^_diff_ = 715.519; df_diff_ = 1, *p* < 0.01), T2 (χ^2^_diff_ = 663.269; df_diff_ = 1, *p* < 0.01), and T3 (χ^2^_diff_ = 528.083; df_diff_ = 1, *p* < 0.01) were all statistically significant, indicating that the two-factor model was the best fitting model at the three time points. These results supported the discriminant validity of the scales at the different measurement time points.

We further examined the factorial invariance across time in both measures (i.e., problem-solving conflict management and trust) separately (see Table 2). The goodness of fit indices of the six baseline models were found to be satisfactory, and all the estimated parameters were statistically significant (*p* < 0.01). MInv1 (structural invariance) had satisfactory fit indices, showing that the problem-solving factor structure and the trust factor structure did not vary across the three measurement waves. These models were compared with three nested more restricted models: MInv2 (invariance of factor loadings), MInv3 (invariance of factor loadings and intercepts), and MInv4 (invariance of factor loadings, intercepts, and measurement errors). MInv2, MInv3, and MInv4 resulted in a satisfactory fit to the data. When comparing these models with MInv1 to test the factorial invariance across time, results revealed that, except for MInv4, χ^2^ differences were not statistically significant. This confirmed the invariance in the factor loadings and item intercepts for both measures (problem-solving conflict management and trust), but not the invariance in the measurement errors or uniquenesses.

**TABLE 2:** Goodness of fit indices for tested invariance models across time.

Model	χ^2^	df	RMSEA	CFI	TLI	Δχ^2^	Δdf
**Problem solving**							
M0a. Baseline model—Problem solving T1	1.645	2	0.000	1.000	1.001		
M0b. Baseline model—Problem solving T2	4.120	2	0.057	0.998	0.994		
M0c. Baseline model—Problem solving T3	5.291	2	0.071	0.997	0.991		
MInv1. Structural invariance—Problem solving	73.376	39	0.052	0.990	0.983		
MInv2. FL. Invariance—Problem solving	82.080	45	0.050	0.989	0.984	8.704	6
MInv3. MInv2 + Intercept Inv.—Problem solving	88.419	51	0.047	0.989	0.986	15.043	12
MInv4. MInv3 + Uniq. Inv.—Problem solving	142.220	59	0.066	0.976	0.973	68.844**	20
**Trust**							
M0a. Baseline model—Trust T1	0.271	2	0.000	1.000	1.007		
M0b. Baseline model—Trust T2	1.864	2	0.000	1.000	1.000		
M0c. Baseline model—Trust T3	6.145	2	0.080	0.995	0.984		
MInv1. Structural invariance—Trust	104.033	39	0.071	0.977	0.961		
MInv2. FL. Invariance—Trust	112.596	45	0.068	0.976	0.965	8.563	6
MInv3. MInv2 + Intercept Inv.—Trust	118.930	50	0.065	0.976	0.968	14.897	11
MInv4. MInv3 + Uniq. Inv.—Trust	145.193	57	0.069	0.969	0.964	41.160**	18

Inv., invariance; FL, factor loadings; and Uniq., uniquenesses. ** *p* < 0.01.

### Hypothesis Testing

Our results indicated that all the models showed satisfactory goodness of fit indices (see Table 3): Model 1 (stability), Model 2 (causality), Model 3 (reversed causation), and Model 4 (reciprocal). Focusing on the model comparison, when comparing the alternative models (Models 2, 3, and 4) with the stability model (Model 1), the results indicated that the inclusion of cross-lagged paths from problem-solving conflict management to trust (χ^2^_M1–M2_ = 24.20; df_diff_ = 3, *p* < 0.01), from trust to problem solving (χ^2^_M1–M3_ = 44.33; df_diff_ = 3, *p* < 0.01), and the reciprocal relations between problem solving and trust (χ^2^_M1–M4_ = 60.38; df_diff_ = 6, *p* < 0.01), offered significantly better fit to the empirical data compared to the model that included only temporal stabilities and synchronous correlations (Model 1). Finally, the results of the χ^2^ difference test in the comparison between the causality model and the reciprocal model (χ^2^_M2–M4_ = 36.18; df_diff_ = 3, *p* < 0.01), and the comparison between the reversed causation model and the reciprocal model (χ^2^_M3–M4_ = 16.05; df_diff_ = 3, *p* < 0.01), showed that the reciprocal model (Model 4) was significantly superior to all the other proposed models, supporting Hypothesis 3.

**TABLE 3:** Fit indices for the hypothesized models.

	χ^2^	df	RMSEA	CFI	TLI
Model 1. Stability model	423.203	219	0.053	0.968	0.960
Model 2. Causality model (Problem solving → Trust)	399.001	216	0.051	0.972	0.964
Model 3. Reversed causation model (Trust → Problem solving)	378.870	216	0.048	0.975	0.968
Model 4. Reciprocal model	362.823	213	0.046	0.977	0.970
Cut-offs	–	–	<0.10	>0.90	>0.90

**FIGURE 1:**
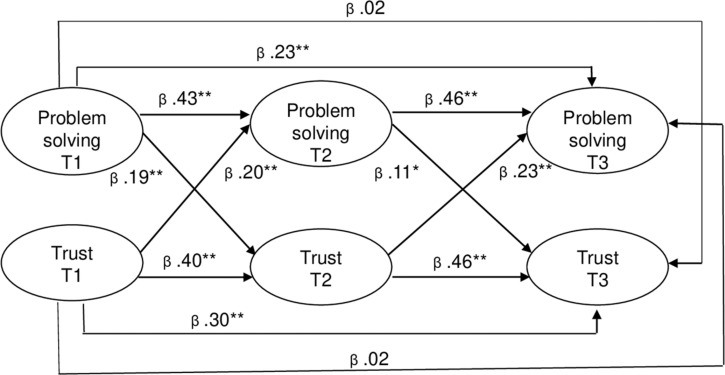
Results for the reciprocal model (model 4). Note: T, Time; *p < 0.05; **p < 0.01 and; one-tailed tests.

Regarding the structural relationships tested in the models, Model 2 resulted in significantly positive lagged effects of T1 problem-solving conflict management on T2 trust (β = 0.23; *p* < 0.01) and of T2 problem solving on T3 trust (β = 0.14; *p* < 0.05), but the structural path from T1 problem solving to T3 trust did not reach significance (β = 0.03; *p* > 0.05). Additionally, Model 3 resulted in significant positive lagged effects of T1 trust on T2 problem solving (β = 0.23; *p* < 0.01) and of T2 trust on T3 problem solving (β = 0.25; *p* < 0.01), but the structural path from T1 trust to T3 problem solving did not reach significance (β = 0.02; *p* > 0.05). Finally, the results of the model that included the reciprocal relationships (Model 4) confirmed the significant cross-lagged effects found in Models 2 and 3 (see Figure 1). These findings provide support for the combination of the causality and reversed models in one complex proposal based on reciprocity, where problem-solving conflict management and trust reinforce each other over time, supporting Hypothesis 3.

### Auxiliary Analyses

In order to rule out the influence of demographic variables such as age, sex, and tenure, we conducted four regression analyses. Our findings revealed that the relationship between trust at T1 and problem-solving conflict management at T2 (*B* = 0.3, *p* < 0.01), and between problem-solving conflict management at T2 and trust at T3 (*B* = 0.44, *p* < 0.01), remains statistically significant after controlling for the professionals’ age, sex, and tenure (years working in the organization). Additionally, the links from problem-solving conflict management at T1 (*B* = 0.40, *p* < 0.01) to trust at T2, and from trust at T2 to problem-solving conflict management at T3 (*B* = 0.54, *p* < 0.01), were also statistically significant after controlling for the effects of age, sex, and tenure in years. Therefore, the results of the regression support the reciprocal model beyond the contribution of professionals’ age, sex, and tenure.

## Discussion

The purpose of the current research study was to test the nature of the interrelations between problem-solving conflict management and trust over time. To do so, we examined four proposals: (a) stability, (b) causality model (problem-solving conflict management contributes to subsequent trust), (c) reversed causation model (trust leads to subsequent problem-solving conflict management), and (d) reciprocal model (a combination of the causality and reversed causation models where problem-solving conflict management and trust are connected in a reciprocal way). Our results confirmed the superiority of the reciprocal model. Accordingly, professionals who perceive that families use problem-solving strategies increase their trust in family members of IID over time, and reciprocally, professionals who trust in families stimulate the use of problem-solving by family members. Furthermore, the cross-lagged effects only appeared for proximal times, but not for distal times. Thus, T1 problem solving was not related to T3 trust, and T1 trust was not related to T3 problem solving.

A relevant contribution of our study is the clarification of the sequence connecting problem-solving conflict management to trust. Most studies have assumed that conflict management strategies are the starting point for developing trust (see Chen and Ayoko, 2012). In other words, scholars assumed that perceiving the social context—how the other party in a relationship manages conflict—is the crucial factor in explaining the emergence of trust. Nevertheless, other scholars have argued that trust is the starting point because this psychological state is able to produce changes in the social context (behaviors of other parties), enhancing problem-solving strategies to manage conflicts (Holtgrave et al., 2020). This debate also exists in other research areas of organizational psychology, such as the investigation of climate. Schneider (1987) complained that the context overly dominates the explanation of human behavior in organizations, and he argued that people are also able to modify the organizational context. Our study is congruent with this rationale, supporting the existence of a complex reciprocal relationship between problem-solving strategies used by others (social context) and trust (personal variable).

Time requires specific attention. Although scholars have called for research that focuses on the dynamics underlying cooperative conflict management and trust (e.g., Tjosvold et al., 2016), there is a lack of studies examining the relationships between the two constructs over time. The predominance of cross-sectional studies provides a static view of how problem solving and trust are related. By contrast, because constructs in organizational psychology evolve over time, the consideration of a dynamic approach provides a richer portrait. Taking into account that time improves our capacity to build theory and realistically capture organizational life (George and Jones, 2000; Roe, 2008), our study exemplifies this perspective by providing empirical evidence of a positive spiral between problem solving and trust over time that static cross-sectional studies could not capture.

Some scholars have argued that research linking cooperative conflict management to trust should go beyond the traditional context of supervisor–employee relationships (e.g., Ferres et al., 2004; Han and Harms, 2010). Because disputes and trust are ubiquitous in organizational life, the consideration of other social interactions (e.g., among team members, between employees, and customers) could help to achieve a more complete view of how problem-solving conflict management and trust are experienced in different contexts. For instance, organizational psychology is increasingly interested in the mutual influence between employees and service users (e.g., Groth and Grandey, 2015), exploring both positive (Zablah et al., 2016), and negative (Dudenhöffer and Dormann, 2013) spirals in their social interactions. With this in mind, we investigated the perspective of professionals who interact with family members in organizations for IID, providing evidence that a positive spiral, characterized by problem-solving conflict management and trust, is possible.

As mentioned above, an important strength of our study is its longitudinal approach. However, as in all research, the present study also has limitations. We stress three shortcomings that provide inputs for future research. First, although it is relevant to consider three measurement times, more waves are welcome in order to achieve a more detailed view of possible temporal trajectories associated with both problem solving and trust. In fact, future studies could examine these trajectories and how changes in one variable are associated with changes in the other. Second, we concentrated on professionals, although families are seen through their “eyes.” Further studies can also consider the perspective of family members explicitly, including their levels of trust and their perceptions of the extent to which professionals use cooperative conflict management, in order to capture the mutual influence in terms of conflict management and trust. Third, our research focused on the problem-solving conflict management strategy. However, the analysis of conflict intensity and conflict type could add value in future studies, providing a more complete picture. For example, it is possible to examine whether the intensity of conflict moderates the relationship between trust and problem solving, or how the (dys)functionality of some conflict management strategies depends on the type of conflict (De Dreu and van Vianen, 2001). Organizational culture oriented toward helping or contributing to others (Pătraş et al., 2018), as part of a broader context, could also help to extend our knowledge about conflict, conflict management, and trust. In addition, distrust, as a separate construct (although related to trust; Vlaar et al., 2007), can be significantly connected to other conflict management strategies.

Despite these limitations, the current study contributes to the knowledge about the dynamic relationship between problem-solving conflict management and trust in professionals who interact with families in organizations for IID. From the perspective of professionals, it is possible to create a positive spiral in the interaction with families. Conflict is not negative in itself, but it might become problematic when not managed properly. The critical issue is to approach it in a cooperative way that fosters reciprocal relationships with trust.

## Data Availability Statement

The raw data supporting the conclusions of this article will be made available by the authors, without undue reservation.

## Ethics Statement

The studies involving human participants were reviewed and approved by Ethical Committee of Research in Humans of the University of Valencia. The participants provided their written informed consent to participate in this study.

## Author Contributions

VM-T led the project, coordinated the preparation of the manuscript, and participated in the wording of the manuscript. YE was the main author and she contributed to the statistical analyses and databases. IT coordinated the statistical analyses and participated in the wording of the manuscript. AM, JR, and LP supported the statistical analyses, participated in the wording of the manuscript. All authors contributed to the article and approved the submitted version.

## Conflict of Interest

The authors declare that the research was conducted in the absence of any commercial or financial relationships that could be construed as a potential conflict of interest.
